# Recapitulation of First Pass Metabolism Using 3D Printed Microfluidic Chip and Organoid

**DOI:** 10.3390/cells10123301

**Published:** 2021-11-25

**Authors:** Bo-Eun Lee, Do-Kyung Kim, Hyunil Lee, Siyeong Yoon, Sin-Hyung Park, Soonchul Lee, Jongman Yoo

**Affiliations:** 1 CHA Organoid Research Center, Department of Microbilogy, School of Medicine, CHA University, 335 Pangyo-ro, Bundang-gu, Seongnam 13488, Gyeonggi-do, Korea; bon5122@gmail.com; 2 Organoidsciences, Ltd., Bundang-gu, Seongnam 13488, Gyeonggi-do, Korea; 3 CHA Bundang Medical Center, Department of Orthopaedic Surgery, School of Medicine, CHA University, Seongnam 13496, Gyeonggi-do, Korea; dokim1018@chauniv.ac.kr (D.-K.K.); tldud1105@naver.com (S.Y.); 4 Department of Orthopaedic Surgery, Ilsan Paik Hospital, Inje University, Goyang 10380, Gyeonggi-do, Korea; I0388@paik.ac.kr; 5 Department of Orthopaedic Surgery, Soonchunhyang University Bucheon Hospital, Bucheon 39371, Gyeonggi-do, Korea; greatsin@empas.com

**Keywords:** first pass metabolism, 3D print, microfluidic, chip

## Abstract

The low bioavailability of oral drugs due to first pass metabolism is a major obstacle in drug development. With significant developments in the field of in vitro organ modeling and microfluidic chip three-dimensional (3D) printing, the challenge is to apply these for the production and evaluation of new drug candidates. This study aimed to produce a microfluidic chip to recapitulate and assess the feasibility of the first pass metabolism. The infill condition of the polycarbonate transparent filament and layer height was optimized to visualize and maintain the organoid or spheroid on the chip. Next, the chip was fabricated using a 3D printer after a computer-aided design (CAD). The chip consisted of three wells of different heights. The small intestinal (SI) organoid and colorectal adenocarcinoma spheroids were placed on the second and third wells, respectively. No additional equipment was assembled, and the tilted tunnel was connected to each well to transport the material by gradient force. The chip was fabricated using 50% and 0.1 um thickness. Among the three different prototypes of chip (chips 1, 2, and 3), the highest distribution of plasmids in the Matrigel of the second well was observed in Chip 2 at 48 h. The effect of first pass metabolism was analyzed using docetaxel. In the chip without an SI organoid, there was a marked decrease in the viability of colorectal adenocarcinoma spheroids due to drug efficacy. However, in the chip with the SI organoid, no significant change in viability was observed because of first pass metabolism. In conclusion, we presented a simple, fast, and low-cost microfluidic chip to analyze the efficacy change of candidate drug by the first pass metabolism.

## 1. Introduction

Oral administration is the most convenient among the different routes of drug administration due to the high patient compliance. However, the extensive effect of first pass metabolism is one of the principal reasons for the poor oral bioavailability of drugs. First-pass metabolism can occur when the drug passes through the intestinal wall and the liver, following oral administration. The role of these organs has been studied extensively. Its extent depends on several physiological factors, including enzymatic activity, plasma protein levels, and gastrointestinal motility [1]. It is a process by which substances are detoxified and converted into their water-soluble forms, promoting easy excretion through the kidneys. However, the required doses of drugs are prevented from entering the systemic circulation due to the metabolism in the intestinal wall and the liver, which results in poor bioavailability and decreased pharmacological activity. Thus, there is a need for higher doses to obtain the minimum effective plasma concentrations. 

Microfluidics is an ever-evolving research field that provides many benefits to biological research, such as reducing the amount of sample and reagents, decreasing the experimental time, and enabling in vivo mimics [2]. Developed in the early 1980s, 3D printing converted computer-assisted design (CAD) into a physical object in a single process [3,4]. Currently, commercial 3D printers capable of producing microfluidics, ranging from a few microns to several centimeters, are beginning to challenge soft lithography as a research prototyping approach to microfabrication [5].

Similarly, advances in cell culture technology enable us to generate 3D in vitro tissue consisting of various cells to mimic the corresponding in vivo organ, which is called an organoid [6]. Organoids offer an advantage over currently used 2D cultures. Specifically, 2D models lack a 3D structure and do not present the complex organization observed in 3D or in vivo structures, thereby lacking the effect of the extracellular matrix on the overall function of the cells. Hence, 2D models show an altered phenotype seen in primary cells and their in vivo counterparts, limiting the translational interpretation of the results. Drug diffusion kinetics are inaccurately modeled in 2D tissue cultures. Drug doses that are effective in 2D culture are frequently ineffective when applied to patients, and the absence of cell–cell and/or cell–ECM interactions in 2D tissue cultures frequently results in the loss of cell function. Instead, 3D tissue architectures perform physiological fluid flow and normal 3D tissue structures. Co-culturing of tumor cells has been reported to result in higher chemoresistance and better metabolization. There is still a need for a system that allows the assessment of efficacy and toxicity changes in libraries of compounds after the compound undergoes first pass metabolism [7].

The current state of drug development models is insufficient for the development of new pharmaceuticals to treat numerous human diseases. There is a need for more precise human-representative systems to simulate the effects of drug candidate compounds on the body. Animal models serve as the gold standard for drug testing, but their disadvantages include high costs and uncertainty in interpreting results in a variety of pathologies. Animal models are not always predictive of human outcomes. In this research, we developed a simple, fast, and cost-effective 3D microfluidic chip for predicting drug efficacy focusing on “first pass metabolism” (FPM chip) and validated the chip’s feasibility using a small intestinal (SI) organoid and a colorectal adenocarcinoma spheroid. The FPM chip used in this study did not use a pump system like other microfluidic chips, so it took less time and was more cost efficient. We have designed an FPM chip that performs physiological fluid flow using the different heights of chip design to media to flow without using the pump. Compared with animal models, fabricated FPM chips represent more physiological conditions for drug metabolism. 

## 2. Materials and Methods

### 2.1. Determination of Basic Condition for 3D Microfluidic Chip (FPM Chip) Fabrication

The model name of the 3D printer was Ultimaker 3 (Ultimaker, The Netherlands), and a polycarbonate (PC) transparent filament was used for chip fabrication because the PC filament has high thermoplasticity and biocompatibility [8]. The device was printed at a speed of 50 mm/s at an AA 0.4 type nozzle and various infill conditions (10%, 30%, 50%, 70%, and 90%). The optimized infill option was determined by calculating the contrast modulation index (Cm) after placing the mouse SI organoids on the chip because image sharpness was essential for cell evaluation. The formula for Cm was as follows: Cm = (Lw − Lb)/(Lw + Lb), where Lw and Lb are the luminance of white color and luminance of black color, respectively. The viability of SI organoids was measured using CellTitier-Glo^®^ 3D assay according to the manufacturer’s instructions on the optimized infill percentage under various layer heights (0.1, 0.15, 0.2 um). All images were acquired using a Nikon ECLIPSE Ti2 (Nikon, Tokyo, Japan).

### 2.2. FPM Chip Design and Automatic Flow Assay

The chip construction was based on an automatic flow without any other electronic supplement equipment in the system. For the automatic flow, the microfluidic chip considered the difference in the height level in each well. The chip consisted of three different wells. The first well was a media inflow or drug injection well, the second well was an organoid culture well, and the third well was another cell culture or media outflow well. Based on this concept, three types of chips were designed using a Fusion 360 (Autodesk, Nashua, NH, USA). They were designed to have the same well size in each part but different connection hole locations between adjacent wells. The flow media included the plasmid vector (pSpCas9(BB)-2A-GFP, Addgen, MA, USA) to determine the flow rate in each chip. Embedded Matrigel (BD Biosciences, Bedford, MA, USA) at the second well, which was the same condition as the organoid culture, and 200 µL of the medium were added to the wells to prevent direct flow out. Moreover, 10 µg plasmid contained media at first well. The media and Matrigel were collected for detection at 48 h and 72 h using a Thermal Cycler Dice^®^ Real-Time System III (Takara, Shiga, Japan).

### 2.3. Mouse SI Organoid

SI tissues were obtained from 6–8 weeks old C57Bl/6 mice (Orient Bio Inc., Seongnam, Korea). The experimental process for animal use was approved by the CHA University Institutional Animal Care and Use Committee. The SI tissue was cut longitudinally from the duodenum to the terminal ileum. It was then sliced into 5–10 mm pieces and washed with cold Dulbecco’s phosphate-buffered saline (DPBS; Welgene, Korea). The crypts were isolated using the Gentle Cell Dissociation Reagent (StemCell Technologies, Durham, NC, USA) for 15 min at 15 rpm shaking and pipetting with dissociation buffer (1% D-sorbitol and 1% sucrose in DPBS). The supernatant was filtered through a 70-µm cell strainer, and the crypts were collected by centrifugation at 270 × *g* for 5 min at 4 °C. The pellet was resuspended in the SI culture medium, mixed with Matrigel at a ratio of 1:1, and incubated with 5% CO_2_ at 37 °C for 10 min. After polymerization of the matrices, the SI culture medium was composed of advanced DMEM/F12 (Gibco, Waltham, MA, USA) with 10% R-Spondin-1 conditioned medium from HA-R-Spondin 1-Fc 293T cells, N2 (Gibco, Waltham, MA, USA), B-27 (Gibco, Waltham, MA, USA), 50 ng/mL mEGF (Peprotech, Rocky Hill, NY, USA), 100 ng/mL mNoggin (Peprotech, Rocky Hill, NY, USA), and 1 mM N-acetylcysteine (Sigma-Aldrich, St. Louis, MO, USA). The culture medium was replaced every 2–3 days. The cultured mSI organoid was embedded in the second well of the FPM chip as previously described.

### 2.4. Human Colorectal Adenocarcinoma (HT-29) Spheroid

HT-29 cells were obtained from ATCC (Manassas, VA, USA). For spheroid formation, 1.0 × 10^6^ cells were suspended in the culture media and mixed with the same volume of Matrigel in a 96-well plate. After 3 days, the spheroid formation was confirmed by microscopy and DPBS washing to remove the spheroids. The spheroids were centrifuged and mixed with 50% Matrigel in culture medium (RPMI-1640 with 10% FBS). The HT-29 spheroids were embedded in the third well of the FPM chip for the simulation assay. 

### 2.5. FPM Chip Simulation Assay

The FPM chip simulation assay was performed as described previously [7]. The mouse SI organoids were placed on the second well, and HT-29 spheroids were placed in the third well of the FPM chip and incubated in a 5% CO_2_ humidified incubator at 37 °C for 20 min. After polymerization, 200 µL of culture media in the second and third well and the media contained various concentrations of docetaxel for 72 h. HT-29 spheroids were collected and dissociated using Trypsin-EDTA 0.25% (Welgene, Korea). The total cell number was counted using a Luna™ cell counter (Logos Biosystems, South Korea), and Adenosine-50-triphosphate (ATP) levels were quantified using CellTiter Glo^®^ 3D (Promega, Madison, WI, USA). Following the simulation assay, the SI organoid and HT-29 spheroids were transferred to a culture plate to confirm the presence of dead cells. Propidium iodide was used to stain the dead cells.

### 2.6. Statistical Analyses

Statistical analyses were performed using GraphPad Prism 5 (GraphPad Software, San Diego, CA, USA). All experiments were performed at least three times. A Mann–Whitney test was used to test the significance when only two groups were tested after the normality test. The Kruskal–Wallis test with post hoc Bonferroni tests was used to test the significance of data in more than two groups. Friedman’s two-way analysis of variance was used in the chip simulation assay. Statistical significance was set at *p* < 0.05.

## 3. Results

### 3.1. FPM Chip Design and Optimization of Basic Condition

**Figure 1:**
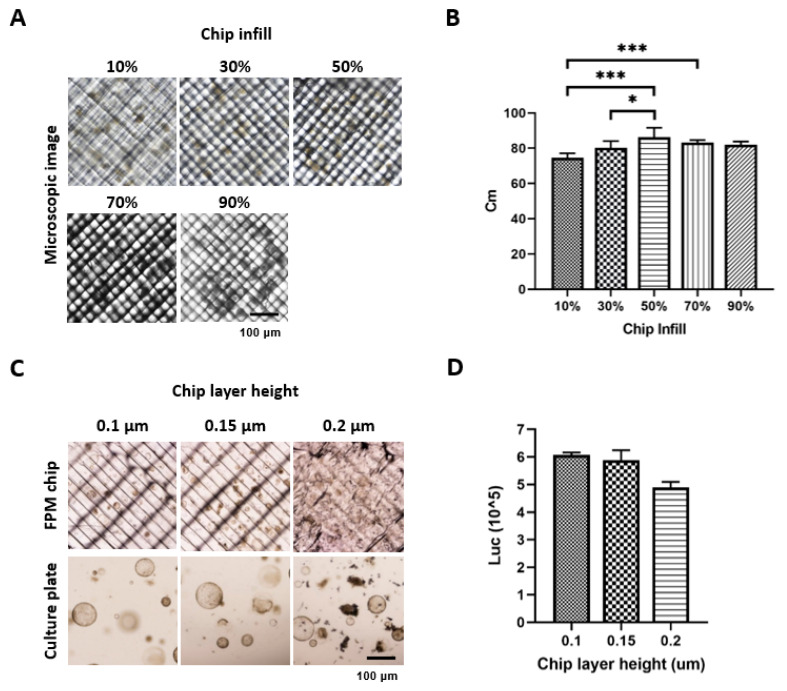
Optimization of the chip infill to visualize the organoid or spheroid under microscopy. Different conditions of the infill (10%, 30%, 50%, 70%, and 90%) were applied to fabricate the chip. Six images of organoid and spheroid on the chip were taken for each condition, and image sharpness was evaluated by calculating the Cm index. An infill condition of 50% showed a significantly higher sharpness compared to any other condition (*p* < 0.05) (**A**,**B**). Next, the organoid viability was measured using CellTiter-Glo^®^ 3D, and the result showed that the 0.1 μm thickness had the highest viability compared to the 0.15 μm and 0.2 μm thickness (**C**,**D**). The differences were considered significant at *p* < 0.05 (*), *p* < 0.001 (***).

First, to take the image during drug screening on the chip, we tried to take the organoid or cell spheroid images using the Nikon ECLIPSE Ti2 (Nikon, Tokyo, Japan) under different infill options. An increased infill percentage resulted in an increased optimization. However, exceeding the acceptable percentage resulted in a thick grid inside the FPM chip, thus a low optimization. The results showed that the 50% infill had the highest resolution of 86.3% of the Cm value (Figure 1A,B). Using a 50% infill option, we measured the organoid viability and found that the 0.1 μm thickness of the FPM chip had the highest organoid viability, although the difference was not statistically significant (Figure 1C,D). 

**Figure 2:**
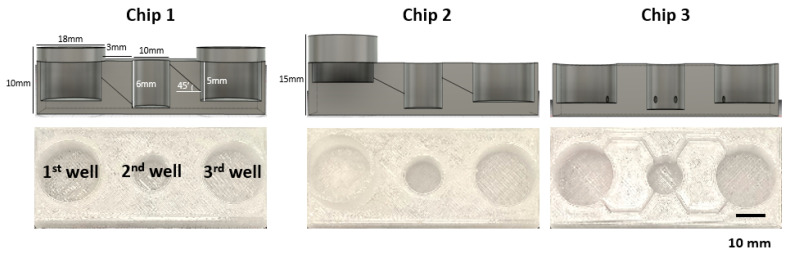
FPM chip design. Using the optimized infill condition, three different types of FPM designs were fabricated. All prototypes had the three wells: first well; media inflow or drug injection well, second well; organoid culture well, third well; another cell culture or media outflow well. Chip 1 had three wells of the same height with the tilted channel for material transfer by gradient force. The three wells of Chip 2 were located at different heights and connected by the tilted channel. Lastly, Chip 3 had the same height wells and the wells were connected by a non-tilted channel.

Next, we designed three different prototypes of the 52 mm × 22 mm sized FPM chip. Chip 1 had three wells of the same size (first and third well: diameter of 18 mm, height of 6 mm. Second well: diameter of 10 mm and height of 6 mm) placed at the same height, and the adjacent wells were connected by a tilted channel. Chip 2 had wells with the same size but located at different altitudes, 5 mm higher than Chip1, which meant that the injection well was located highest in the chip, the organoid culture well was halfway between the injection well and the outflow well, and the outflow well was the lowest among the wells. A tilted channel connected the adjacent wells. Chip 3 had three wells of the same size placed at the same height, and the adjacent wells were connected by a non-tilted channel (Figure 2).

### 3.2. Plasmid Distribution Test

**Figure 3:**
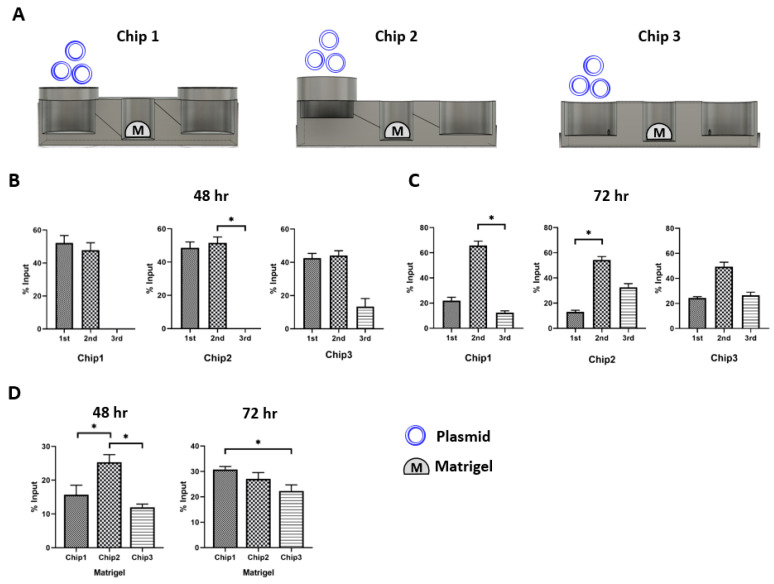
Plasmid distribution test. The plasmid was injected in the first injection well, and the distribution of plasmid was measured at 48 h and 72 h after locating the organoid in the second well. In Chip 2, The Matrigel had the highest plasmid concentration in Chip 2 (*p* < 0.05). The only scant amount of plasmid was counted in the third well at 48 h. Then, the plasmid in the second well was transferred to the third well at 72 h. There was no specific difference in the pattern of plasmid distribution at 72 h among the three chip prototypes. Scheme of the simulation assay (**A**), plasmid distribution after 48 h (**B**) and 72 h flow (**C**), comparison of plasmid concentration in the Matrigel of second well at 48 h and 72 h (**D**). The differences were considered significant at *p* < 0.05 (*).

To evaluate the design compatibility of the three FPM chip prototypes in the recapitulation of the first pass metabolism, we placed Matrigel in the second well (Figure 3A). Then, 10 µg plasmid was added to the first injection well and the plasmid distribution in the three different wells was measured at 48 h and 72 h after plasmid treatment (Figure 3B,C). The results showed that Chip 2 had the highest plasmid concentration in the Matrigel at 48 h compared to the other FPM chip prototypes. Chip 3 had the lowest plasmid concentration in Matrigel (Figure 3D). Interestingly, both prototypes 1 and 2 had a small plasmid in the third outflow well at 48 h. However, we observed the plasmid movement toward the third outflow well at 72 h, and there was no significant difference in the plasmid distribution among the three chips at 72 h. Moreover, Chip 2 with the infill of 50% and 1 µm thickness was the most compatible to test the effect of first pass metabolism.

### 3.3. First Pass Metabolism Simulation on Chip

Finally, to further explore the feasibility of the FPM chip for the first pass metabolism, various anti-cancer drug concentrations were treated in the first well after mSI organoids and HT-29 spheroids were placed in the second and third wells, respectively. We used docetaxel and paclitaxel because these drugs are not orally administered due to first pass metabolism, which may lead to the drug’s dissolution in acidic gastric fluid and permeation across the enterocytes by cytochrome P3A4.

**Figure 4:**
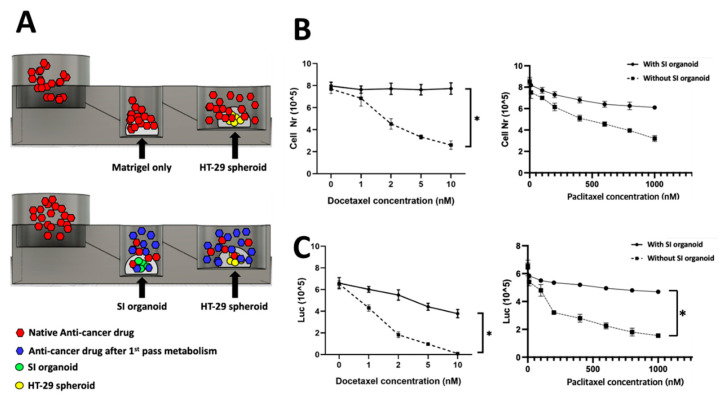
Chip simulation assay. Anti-cancer drug was treated in the first well with or without an SI organoid in the second well. For the control, only Matrigel was placed instead of the SI organoid in the second well. The spheroids’ viability in the third well was measured to validate the working of the FPM chip. When Docetaxel was used to treat the SI organoid, there was no specific change of the spheroids’ viability in the third well, but the Docetaxel without the organoid significantly decreased the viability at 72 h. Scheme of the simulation assay (**A**), total cell number and metabolic activity assay using CellTiter Glo^®^ 3D after 72 h flow in Docetaxel (**B**) and Paclitaxel (**C**). The differences were considered significant at *p* < 0.05 (*).

After 72 h of treatment, the total number of live cells was used to measure the viability of HT-29 spheroids. In the chip with SI organoids, the viability of HT-29 spheroids in the third well was not significantly decreased despite an increase in the drug concentration, which meant that the anti-cancer drug was metabolized in the SI organoid of the second well. The drug efficacy was decreased. However, there was a significant change in HT-29 spheroid viability in the chip with the SI organoid, and the effect was proportional to the drug concentration. The cell number in the chip with SI organoid decreased slightly by 8.2% (*p* > 0.05), while the cell number in the chip without SI organoid was substantially reduced by 76.5% at 72 h (*p* < 0.01) (Figure 4B).

Metabolic activity was evaluated using an ATP colorimetric/fluorometric assay. The ATP levels of colorectal adenocarcinoma spheroids were measured with or without SI organoids. The results showed a trend similar to that of cell viability. Additionally, the ATP level of HT-29 spheroids was significantly decreased in the presence of SI organoids (*p* < 0.01) (Figure 4C).

**Figure 5:**
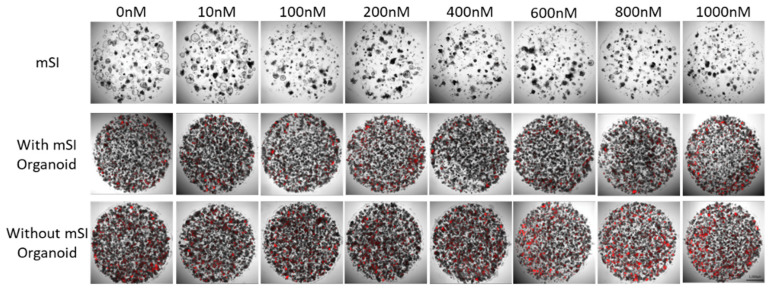
Dead cells labeled using PI (red) in mSI organoid and HT-29 spheroid after anti-drug treatment for 72 h.

The confirmation of anti-cancer drug toxicity in SI organoids, organoids, and HT-29 spheroid cells was carried out on a cell culture plate using propidium iodide staining. The number of dead HT-29 spheroid cells increased at 600 nM. The SI organoid did not have any dead cells, but it continued to grow (Figure 5).

## 4. Discussion

In this study, we demonstrated the feasibility of a microfluidic chip that recapitulates the first pass metabolism. First, we optimized the FPM chip using different 3D printing conditions. Then, we validated its reproducibility using the well-known anti-cancer drug docetaxel, which is metabolized in the SI and then activated.

With the great advances in mimicking organ physiology, the implementation of these techniques for drug candidate and compound evaluation in a routine setting is still unclear. Therefore, there is a need for a system that allows the assessment of the efficacy and toxicity effects of library compounds. Critical prerequisites of such a platform include compatibility with automated high-content imaging equipment and relatively fast readouts with lower cost. Additionally, the model should utilize limited amounts of cell material per data point, while still mimicking the complexity of human organs to the extent necessary for acquiring physiologically relevant responses [9]. 

Researchers have attempted to recapitulate the first pass metabolism for drug development, including co-culture and animal experiments [10]. However, these 2D/3D or direct/indirect co-culture systems cannot necessarily reflect the specific characteristics of organs and are technically demanding and time consuming. In addition, reproducibility has not been determined in the screening of candidate drugs [11].

Animal experiments are mandatory before clinical trials because existing in vitro strategies and methods cannot imitate the complex human microenvironment. Cells grown in vitro are in a static macroscale environment that is completely different from the biological environment of the cells grown in vivo [10]. Studies have advocated in vitro/in vivo discrepancies [12,13,14]. However, the animals’ pain, distress, and death during scientific experiments have been a debating issue for a long time. Besides the primary concern of ethics, animal experimentation has several disadvantages, such as a skilled workforce, time-consuming protocols, and high costs [15].

Moreover, the results of the in vivo experiment did not necessarily suggest clinical efficacy in human beings because human beings have hierarchical systems, from the subcellular level to the whole body [16,17]. Consequently, animal data are not being translated to successful clinical outcomes, as evident from failure rates of over 90% between nomination for phase I clinical trials and approval of new drugs [18]. In this context, developing an in vitro drug screening model that mimics live human conditions with high efficiency is one of the challenges that researchers face today.

The attraction of 3D printers is two-fold. The first is their unprecedented ability to fabricate in 3D in a way that has not been previously possible. This technique presents new opportunities in the field of microfluidics, as researchers begin to imagine what might be possible when manipulating surfaces and fluids in 3D. The second feature is the ability to rapidly realize a model, which enables researchers to adopt a “fail fast and often” strategy [5].

We validated our chip using docetaxel, a semisynthetic taxane (Figure 4) prepared by the chemical modification of an inactive precursor from the needles of the European yew tree (*Taxus baccata*) [19]. It acts by disrupting the microtubule network that blocks cell cycles in the late G2 and M phases, thus inhibiting cell replication. The drug has clinically significant antitumor activity against a range of tumor types. It is approved to treat breast cancer, ovarian cancer, non-small cell lung cancer, head and neck carcinoma, gastric cancer, and prostate cancer. This drug is well known not to be administered through the oral route because of first pass metabolism, which leads to the dissolution in acidic gastric fluid and permeation across the enterocytes by cytochrome P2A4 [7].

Our chip had several advantages. We used the SI organoid rather than the 2D culture model. Organoids have several advantages over traditional 2D cultures or animal studies. First, an organoid is easy to handle and can be used for long-term culture as a cell line. Second, organoids have the characteristics of multi-cellular tissue proxies, which recapitulate the cell-to-cell physiology shown in the live human body more precisely. Third, it is feasible to generate a patient-derived specific disease model. Finally, organoid cultures can be started from small tissue samples, typically obtained by biopsy or from surgical specimens. No purification of stem cells is necessary [20]. Our results also demonstrated that this organoid could reflect SI characteristics, particularly the first pass metabolism, and could be fixed in the chip without significant movement by the media flow. Second, we did not need additional equipment, such as an electronic pump, because our chip was designed to distribute the media in the SI organoid efficiently by gravity. Third, this chip was inexpensive and easy to produce using a conventional 3D printer.

This study had several limitations. First, we did not test the reproducibility of the FPM chip using the liver, which is a crucial organ in the first pass metabolism of drug development. Instead, we used an SI organoid, which is the most popular type of organoid. Second, it would still be necessary to optimize our chip fabrication because we did not test it using different materials.

In conclusion, the microfluidic chip developed in this study should be used to test the first pass metabolism of candidate drugs quickly and cost-effectively.

## Data Availability

Data is contained within the article.

## References

[1] Pond S.M., Tozer T.N. (1984). First-pass elimination basic concepts and clinical consequences. Clin. Pharmacokinet..

[2] Aziz A.A., Azmi M.A.M., Zulkifli M.N., Nordin A.N., Bahrain A.K., Makmon F.Z., Badruzzaman N.A., Sabdin S.J.M.T.P. (2019). Rapid fabrication and characterization of PDMS microfluidics device using printed conductive silver ink. Mater. Today Proc..

[3] Becker H. (2009). Hype, hope and hubris: The quest for the killer application in microfluidics. Lab Chip.

[4] Gao W., Zhang Y., Ramanujan D., Ramani K., Chen Y., Williams C.B., Wang C.C., Shin Y.C., Zhang S., Zavattieri P.D. (2015). The status, challenges, and future of additive manufacturing in engineering. Comput. Aided Des..

[5] Waheed S., Cabot J.M., Macdonald N.P., Lewis T., Guijt R.M., Paull B., Breadmore M.C. (2016). 3D printed microfluidic devices: Enablers and barriers. Lab Chip.

[6] Olayanju A., Jones L., Greco K., Goldring C.E., Ansari T. (2019). Application of porcine gastrointestinal organoid units as a potential in vitro tool for drug discovery and development. J. Appl. Toxicol..

[7] Park E., Kim H.K., Jee J., Hahn S., Jeong S., Yoo J. (2019). Development of organoid-based drug metabolism model. Toxicol. Appl. Pharmacol..

[8] Bhattacharjee N., Urrios A., Kang S., Folch A. (2016). The upcoming 3D-printing revolution in microfluidics. Lab Chip.

[9] Wevers N.R., Van Vught R., Wilschut K.J., Nicolas A., Chiang C., Lanz H.L., Trietsch S.J., Joore J., Vulto P. (2016). High-throughput compound evaluation on 3D networks of neurons and glia in a microfluidic platform. Sci. Rep..

[10] Marimuthu M., Kim S. (2011). Microfluidic cell coculture methods for understanding cell biology, analyzing bio/pharmaceuticals, and developing tissue constructs. Anal. Biochem..

[11] Hirsch C., Schildknecht S. (2019). In vitro research reproducibility: Keeping up high standards. Front. Pharmacol..

[12] Schug M., Stöber R., Heise T., Mielke H., Gundert-Remy U., Godoy P., Reif R., Blaszkewicz M., Ellinger-Ziegelbauer H., Ahr H.J. (2013). Pharmacokinetics explain in vivo/in vitro discrepancies of carcinogen-induced gene expression alterations in rat liver and cultivated hepatocytes. Arch. Toxicol..

[13] Ellinger-Ziegelbauer H., Gmuender H., Bandenburg A., Ahr H.J. (2008). Prediction of a carcinogenic potential of rat hepatocarcinogens using toxicogenomics analysis of short-term in vivo studies. Mutat. Res..

[14] Beekman J.M., Boess F., Hildebrand H., Kalkuhl A., Suter L. (2006). Gene expression analysis of the hepatotoxicant methapyrilene in primary rat hepatocytes: An interlaboratory study. Environ. Health Perspect..

[15] Doke S.K., Dhawale S.C. (2015). Alternatives to animal testing: A review. Saudi Pharm. J..

[16] Bale S.S., Moore L., Yarmush M., Jindal R. (2016). Emerging in vitro liver technologies for drug metabolism and inter-organ interactions. Tissue Eng. Part B Rev..

[17] Olson H., Betton G., Robinson D., Thomas K., Monro A., Kolaja G., Lilly P., Sanders J., Sipes G., Bracken W. (2000). Concordance of the toxicity of pharmaceuticals in humans and in animals. Regul. Toxicol. Pharmacol..

[18] Plenge R.M., Scolnick E.M., Altshuler D. (2013). Validating therapeutic targets through human genetics. Nat. Rev. Drug Discov..

[19] Sohail M.F., Rehman M., Sarwar H.S., Naveed S., Salman O., Bukhari N.I., Hussain I., Webster T.J., Shahnaz G. (2018). Advancements in the oral delivery of Docetaxel: Challenges, current state-of-the-art and future trends. Int. J. Nanomed..

[20] Drost J., Clevers H. (2017). Translational applications of adult stem cell-derived organoids. Development.

